# Phosphorus retention in calcareous soils and the effect of organic matter on its mobility

**DOI:** 10.1186/1467-4866-7-6

**Published:** 2006-06-12

**Authors:** Ray von Wandruszka

**Affiliations:** 1 Department of Chemistry, University of Idaho, Moscow, ID 83844-2343, USA

## Abstract

A survey of the interactions between phosphorus (P) species and the components of calcareous soils shows that both surface reactions and precipitation take place, especially in the presence of calcite and limestone. The principal products of these reactions are dicalcium phosphate and octacalcium phosphate, which may interconvert after formation. The role of calcium carbonate in P retention by calcareous soils is, however, significant only at relatively high P concentrations – non-carbonate clays play a more important part at lower concentrations. In the presence of iron oxide particles, occlusion of P frequently occurs in these bodies, especially with forms of the element that are pedogenic in origin. Progressive mineralization and immobilization, often biological in nature, are generally observed when P is added as a fertilizer.

Manure serves both as a source of subsurface P and an effective mobilizing agent. Blockage of P sorption sites by organic acids, as well as complexation of exchangeable Al and Fe in the soil, are potential causes of this mobilization. Swine and chicken manure are especially rich P sources, largely due the practice of adding the element to the feed of nonruminants. Humic materials, both native and added, appear to increase recovery of Olsen P. In the presence of metal cations, strong complexes between inorganic P and humates are formed. The influence of humic soil amendments on P mobility warrants further investigation.

## Background

The mobility of phosphorus (P) in the shallow subsurface is a matter of critical importance and considerable complexity. Its importance stems from the fact that P, an essential nutrient for all plant and animal life, is often in short supply. Agricultural fertilizers and other soil amendments, such as mineral P fertilizers and animal manure, provide P that is readily available to plants. The short-term availability of P to crops is strongly influenced by biochemical processes that affect organic matter, while its long-term status is generally determined by geochemical transformations.

The nature of P species in the shallow subsurface varies widely with location, soil type, and management system. In describing P movement in soils, workers often use operational categorizations such as "dissolved reactive P", "particulate unreactive P", *etc*. [1,2] The abundances of the principal P compounds, expressed as percentages of total P in the soil, are typically in the ranges: orthophosphates 60 – 80%; pyrophosphate 0.5 – 4%; P-monoesters 16 – 38%; and P-diesters 1.2 – 4% [3]. Both inorganic P (P_i_) and organic P (P_o_) species interact extensively with soil components and are subject to various chemical transformations that affect the retention of the element.

Depletion and oversupply are the two main challenges in subsurface P management. Depletion is especially serious when low input agriculture is practiced, involving land clearing and continuous cultivation that reduce both P_i _and P_o _in the soil [4]. Oversupply occurs when amendments are added in excess of crop requirements, as may happen when manure is applied to satisfy the nitrogen requirements of crops [5,6]. Surplus P can be transported in runoff after rainfall, irrigation and snowmelt, and may contribute to eutrophication in water bodies.

The monitoring and management of environmental P is predicated on accurate determinations of the element in subsurface matrices. A thorough discussion of P analysis is beyond the scope of this survey, but an excellent compilation of analytical techniques has been published under the auspices of the USDA-CREES [7]. Sample treatment methodologies for a wide range of environmental samples have been reviewed by Worsfold *et al*. [8]. Briefly, three techniques are widely used for environmental P determination:

(i) The Murphy-Riley (MR) colorimetric method for inorganic P analysis [9] (later improved by Harwood *et al*. [10]), which uses ammonium molybdate, ascorbic acid, and antimony potassium tartrate to develop a blue color with P_i _(absorption at 880 nm) [11].

(ii) Inductively coupled plasma (ICP) spectroscopy, with either optical emission (178.290 nm) [12] or mass spectrometric detection [13]. ICP generally yields higher P values than MR.

(iii) Potentiometry with the phosphate-sensitive cobalt electrode, which was introduced by Xia *et al*. in 1995 [14,15], and has since proven to be a useful sensor for dissolved orthophosphates [16-18].

Chemical identification or organic P in environmental samples is generally carried out by ^31^P NMR spectroscopy [19,20]. Spectral assignments can be challenging, and Turner *et al*. [21] have published extensive lists of P resonance peaks that provide a guideline for the identification of both P_i _and P_o_. Cade-Menun *et al*. note that the quantitative use of ^31^P NMR spectra of soil and litter extracts in solution requires careful sample treatment, control of parameters, and knowledge of the species present in solution. They have published a comprehensive summary of recommendations regarding the choice of extractant, measurement of relaxation times, determination of Fe and Mn content, use of appropriate delay times, and sample temperature [22].

### P in calcareous soils

Analyses of P retention and mobilization in natural calcareous environments have shown that both adsorption and precipitation take place, although it is not always easy to distinguish between the two mechanisms. Measurement of P by any of the techniques mentioned above is usually preceded by single or sequential extractions, which often involve the solvent systems summarized in Table 1.

**Table 1:** Extraction of Inorganic P from Soils*

Extractant	P forms extracted
NaOH/NaCl	Al- and Fe-bound
Na citrate-bicarbonate (CB)	Labile pedogenic Ca-rich
Na citrate (C)	Pedogenic Ca-phosphates
Na citrate-ascorbate (CA)	Occluded in poorly crystalline Fe-oxides
Na citrate-bicarbonate-dithionite (CBD)	Occluded in crystalline Fe-oxides
Na acetate	Ca phosphates (excl. lithogenic apatite)
HCl	Lithogenic apatite

*adapted from ref. [23]

Early work by Cole *et al*. on the interactions of P with calcite surfaces [24] distinguished between initial adsorption and subsequent precipitation of dicalcium phosphate (DCP, CaHPO_4_). Alternatively, octacalcium phosphate (OCP, Ca_8_(HPO_4_)_2_(OH)_2_), may be formed [25], and hydrolytic conversion from DCP to OCP is known to take place [26]. This is especially noted when the initial DCP formation is followed by an amorphous-to-crystalline transition in the solid phase [27]. A cyclic process, in which OCP disproportionates to reform DCP and stable hydroxyapatite (HAp, Ca_10_(PO_4_)_6_(OH)_2_), has also been found to occur [28]. The general consensus, affirmed by X-ray diffraction [29], is that P growth on a calcite surface involves both DCP and OCP, with the former dominating. Surface coverage, even at high P concentrations is typically no greater than 5%.

Early studies with limestone particles [30] suggest that the solubility of P in suspensions of these is also controlled by a DCP solid phase, despite some inconsistencies in solubility product values [31]. The initial attachment involves chemisorption of P onto the particles, producing a material was first thought to be amorphous [27], but was later shown to have a well defined chemical structure [32]. The initial chemisorption, involving the formation of DCP, is described by a Langmuir isotherm, while subsequent sorption (including the formation of OCP) is of a low-energy physical type [33]. The initial process is relatively rapid, followed by a *ca*. 2-h induction period, and then followed by precipitation [34]. P-sorption on all carbonates strongly depends on surface characteristics, especially surface area and zeta potential (17.7 – 25.3 mV). Interestingly, pyrophosphate does not interfere with P sorption, but does appear to inhibit precipitation.

It is clear that surface adsorption and precipitation are major mechanisms of P retention in calcareous systems, depressing its availability after fertilizer application. Diverse results have been obtained regarding the relative roles of carbonates and oxide clays in P retention in calcareous soils. Afif *et al*. [35] found that at high application rates, P available to plants is negatively correlated to the amount of lime in soil, but not to Fe, clay content, or CEC. In contrast, other studies indicate that P retention increases with the ratio of Fe oxides to CaCO_3 _[36,37]. The preponderance of the evidence [38-40] suggests that non-carbonate clays provide most of the P adsorbing surfaces in many calcareous soils, especially at low P concentrations. It has even be reported that a 1.6% (w/w) coating of Fe_2_O_3 _on calcite increases the P sorption capacity 9-fold [41]. As the P content of the soil increases, sorption by carbonates becomes more important.

In comparing the relative importance of surface reactions and precipitation in P retention, Tunesi *et al*. [42] concluded that in soils with a high reservoir of exchangeable cations, precipitation is the predominant mechanism in the reduction of available P. HAp is the most stable precipitated form of P in calcareous soils [43], while other forms, including DCP dihydrate, OCP, and a metastable phase of HAp [44], are somewhat more soluble.

A third retention mechanism for P, especially iron rich soils, involves occlusion in Fe oxide particles. From data obtained from single and sequential extractions with ascorbate, citrate-ascorbate, bicarbonate, dithionite, and oxalate, Torrent and coworkers [23,45] concluded that poorly crystalline Fe oxides (primarily ferrihydrite) have a distinct tendency to occlude P. Reduction of these particles in aquatic environments can lead to increased P concentrations. It has been shown that the relative quantities of P occluded in both poorly and highly crystalline Fe oxides is not necessarily related to the degree of P enrichment in the soil, and that this form of P may in fact be pedogenic in nature [46]. The typical breakdown of P for such a case is shown in Table 2.

**Table 2:** Quantities of P Extracted from Calcareous Marsh Soil*

Types of P in soil (mg/kg)							P by sequential fractionation (mg/kg)						
Total	Inorganic	Organic	Residual	Olsen	P_cb_	P_d_	NaOH	CB	CC	CA	CBD	NaOAc	HCl
839	611	122	105	26	111	145	6	127	389	82	37	13	60

Pcb = CB extr; Pcbd = CBD extr.; ca = citrate-ascorbate; CB = citrate-bicarbonate; CBD = citrate-bicarbonate-dithionite; CC = citrate (2extr).

*adapted from ref. [46]

From a practical standpoint it is interesting to consider how P interactions in calcareous soils compare to those in limed acid soils. In cases where substantial amounts of metal phosphates have accumulated in soils of both types – due to pedogenesis and/or fertilizer application in excess of plant uptake – calcareous soils are found to contain less surface P than limed acid soils. Ca phosphates predominate in the former, and Al and Fe phosphates in the latter. Overall, P availability to plants is greater in limed acid soils [47].

In unamended soils, especially those not having received manure, P leaching is generally a relatively minor problem compared to erosive losses of the element. There is, however, strong evidence that the extent of subsurface P loss is closely related to the degree of phosphorus saturation (DPS) of the soil. In terms of Olsen P, it has been suggested that below 60 mg P kg^-1^, P_i _is sorbed strongly, while at higher concentrations sorption energy is much lower [48], which would promote P leaching. It is generally found that at DPS levels below 20% P leaching is rather insignificant, but increases rapidly above this value [49,50]. When manure is added to soils, however, the situation changes radically, and P is mobilized and subject to both surface and subsurface losses [51,52]. This is further discussed below.

### Influence of organic matter

Both added manure or litter and native organic matter (humic materials) have significant effects on subsurface P retention. Manure not only affects sorption and precipitation of P, but often contains significant amounts of the element, which is thereby – deliberately or incidentally – added to the land. Humic materials, the breakdown products of the total biota in the environment, generally are not a major source of P, but they do have a mobilizing effect on it in the subsurface. The use of extrinsic humates, especially leonardite humic acid, for soil improvement has experienced an upswing in recent years [53].

#### Manure

The application of manure is widely practiced to increase the productivity of soils that contain inadequate levels of organic carbon. The effects of manure on P availability in various soils has been widely studied, and the general conclusion has been that it is a source of P; interacts with soil components in a manner that increases P recovery by crops; and enhances the effectiveness of inorganic P fertilizer. P added from manure and other sources, however, tends to become less available to plants with the passing of time [54]. As mentioned above [5], manure application guidelines are frequently based on the N requirements of crops, and P is therefore often oversupplied and liable to either accumulate or be removed by surface or subsurface transport [55]. As regards the eventual status of fertilizer P in soil, it is interesting to note that manure and mineral (KH_2_PO_4_) fertilizer appear to contribute to different P pools [56]. The latter is efficient at increasing CaCl_2 _extractable P and Mehlich-3 P, while manure (especially chicken manure) has a greater effect on modified Morgan P, as well as other types of P.

Alkaline soils subject to long-term manure amendments have been shown to accumulate substantial quantities of P, with 50–66% in plant available forms [5]. Irrigated plots receiving high (>60 Mg ha^-1^) annual manure applications are considered to pose a risk of ground water contamination, as the total P concentration increases with soil depth. The ability of acid soils to retain added P after long-term manuring, is generally low. It has been reported that manure applications have a greater effect on the retention of P_i _than the retention of P_o _[57].

The affinity constants and sorption capacities of soils for P are reduced by organic amendments, especially manure. This can be due to competition for P fixation sites by organic acids, and/or the complexing of exchangeable Al and Fe by components of manure [58-60]. The latter may, at least partially, be ascribed to the release of sulfates and fluorides by the manure, both of which are strong complexing agents for Al and Fe.

Parallels may be drawn between the P mobilizing effects of manure and humic materials (*vide infra*) on the one hand, and root exudates on the other hand. It is well established that cover crops such as white lupin (*Lupin albus L*.) form cluster roots in response to P deficiency, and that these root systems are efficient producers of succinate, citrate, and malate [61,62]. Release of these organic anions into the rhizosphere enhances the release of sparingly soluble P, not only from the acid soluble pool, but also from more stable residual P fractions. Little information is presently available on the chemical nature of analogous chemical species in manure and humic amendments that may be responsible for their P mobilizing qualities.

On a seasonal basis, a decrease in soluble P during the growing season is often observed in calcareous soils, followed by an increase in the noncropping season [63]. Vivekanandan and Fixen [64] have reported that large-scale manure applications to a silty clay loam results in a linear increase in available P (Bray P1), up to a (presumably) soil dependent limit. P stabilization eventually occurs through apatite precipitation. In acidic soils, high application rates of manure also lead to P mobilization, indicating that organic materials with high P content may substitute for CaCO_3 _as a soil amendment to decrease the P sorption capacity and increase the pH [60,65]. Interestingly, it has recently been reported that dissolved organic matter does not inhibit P sorption in highly weathered acidic soils [66].

#### Types of manure

The type of manure used for soil amendment is an important variable with respect to the amount of P contributed to the soil. Sharpley and Moyer [67] have published a detailed account on the P content of dairy, poultry, and swine manures, both raw and composted. In all cases listed, it was found that P_i _constitutes the vast majority of P determined. Some of the results are summarized in Table 3, which also includes data on P mobilized by simulated rainfall.

**Table 3:** P in Manures**

	Dairy manure	Poultry manure	Swine slurry
total P (mg/kg)	3,990	28,650	32,950
% inorganic P	63	84	91
% organic P	25	14	8
% residual P	12	2	1
%P leached in rainfall*	58	21	15

* 5 consecutive simulated rainfall events of 70 mm/h, 30 min each.

**adapted from ref. [67]

All commercial animal production can cause serious manure disposal problems, which have been exacerbated by extensive consolidation in recent years. The vast quantities of manure [68] produced by centralized pig farming, for instance, are a case in point. The relative amount of P contained in this manure is large, because pigs (and other nonruminants) lack the phytase [69] enzymatic system that releases P from phytic acid stored in cereals. Animal feed producers and farmers therefore often add P_i _to the feed, which improves animal health but also increases the P content of manure. Other approaches in supplying P to pigs include the addition of phytase to the feed, and even the development of phytase transgenic pigs [70] – which, to date, do not appear to have found commercial application. Also, low-phytate corn [71] and barley [72] mutants, usable as feed, have been isolated. Leytem *et al*. [73] found that pigs that were fed these grains showed evidence of hind-gut hydrolysis of phytic acid, possibly by intestinal microflora.

Pig slurry, which is 5–10% solid matter, typically contains 1–2% (dry w/w) P. The bulk of this (75–85%) is P_i_, consisting of CaHPO_4_·2H_2_O and apatites of low solubility [74]. Short term (24 h) adsorption experiments in sandy soils have shown that the average sorption capacity is about 10 kg P_i_/ha·cm depth, so that for every cm of soil a total of 8–12 tons/ha of slurry with ca. 8% solid content can be applied before saturation sets in and mobility increases. Gerritse notes [74], however, that saturation is temporary and is followed by a phase transition (mineralization) that leads to long term immobilization.

The chemical identification of P species in manure is of considerable practical importance, since the exact nature of P is a major determinant in the subsurface retention of the element after manure application. Work by Crouse *et al*. [75] has shown that the mineralization of P_o _by phosphatase enzymes, especially phosphomonoesterase, can proceed over periods extending to 20 weeks in soils amended with chicken manure. The orthophosphate content of the soils increases during mineralization, while P_o _decreases. The sorption of P_o _(nucleotides and inositol hexaphosphate, IHP) is positively correlated with both organic matter and Fe and Al content of the soil [76]. Especially IHP is strongly retained.

The physico-chemical characteristics of manure differ from those of soil, and the use of sequential extractions in manure analysis needs to be carefully evaluated. [77] A major portion of P in manure is soluble in weak extractants such as H_2_O and NaHCO_3_, while much of the soil P requires NaOH and HCl. This is related to the fact that soils contain *ca*. 15 times as much Al, and 10 times as much Fe as manure, while manure tends to have higher Ca and Mg contents. Rapid evaluation of plant-available P clearly is a desirable feature of subsurface analysis, and He and coworkers have introduced a shortcut in the assessment of contributions from manure amendments. They suggest that a single P extraction from dairy manure with a 100 *mM *acetate buffer at pH 5.0 equals the combined H_2_O, NaHCO_3_, and NaOH extractions [78].

Turner and Leytem caution that the presence of organic P in the HCl extract of the Hedley fractionation [79] procedure is commonly overlooked, resulting in under-reporting [80]. They found phytic acid to be present in HCl extracts of broiler litter and swine manure, indicating that this relatively immobile compound enters the environment from these sources. More mobile P_o _species in manure, such as phospholipids and simple phosphate monoesters, can, despite their relatively low abundance, become a major P component in runoff [81]. Turner and Leytem also introduced a two-step fractionation procedure for manure P [80], involving 0.5 M NaHCO_3 _for readily soluble P, followed by 0.5 M NaOH/50 mM EDTA for more recalcitrant P. Recoveries were superior to those obtained with Hedley and NaHCO_3_/HCl procedures.

P_i _in the H_2_O extractable fraction of dairy manure is correlated with total P (P_o _is not [78]), while the opposite is true for the NaHCO_3 _extractable fraction. About half of the P_o _in the H_2_O fraction is enzymatically hydrolysable – mainly as phytate in pig manure [82]. In contrast, a major portion of P_o _in the NaHCO_3 _fraction is not hydrolysable by either wheat phytase, alkaline phosphatase, nuclease P1, or nucleotide pyrophosphatase. This indicates that P_o _extracted from manure with NaHCO_3 _is not especially labile.

#### Manure treatment

Chicken manure and swine slurry are apt to provide readily mobile (water soluble) P to soil, which can lead to runoff and eutrophication problems. For this reason, some effort has been expended at reducing the mobility of P in these types of manure and litter. Chemical additives that have been used for this purpose [83,84], include lime, ferric chloride, ferrous sulfate, and alum (Al_2_(SO_4_)_3_·14H_2_O or KAl(SO_4_)_2_·12H_2_O). Of these, alum has proven to be most effective, with the added benefit that it also prevents the loss of ammonia [85,86] and water soluble metals from manure amended soils [87,88].

Al-associated P accounts for some 40% of total P in alum amended materials, with about 20% of this being drawn from Ca-phosphate phases. This decreases by about half when alum is added to poultry litter. Hunger *et al*. [89] have used ^31^P NMR to elucidate the nature of the immobilized P species. This proved to be a difficult task, involving many unresolvable P_i _and P_o _species. It was noted that no crystalline aluminum phosphate species were present.

#### Humic materials

Humic and fulvic acids comprise a wide variety of organic materials that are present in all agricultural soils. Their effects on plant growth and nutrition are well documented, [90,91] and they can be applied to improve soil structure and increase crop yields. Reports on the influence of humic materials on P retention and release have largely focused on the mineral components of the soils studied. Recent work indicates that the occurrence of Al and Fe has a significant effect on the P sorption capacity, despite the presence of large amounts of organic matter [92]. Earlier, it had been shown that P decreases the sorption of organic C to acid mineral soils, suggesting a ligand exchange process at the surface [93,94]. As regards the reverse, *i.e*. the release of P under the influence of dissolved humic materials, Delgado *et al*. [95] have published one of the few accounts dealing with this issue. They found that application of humics to the soil increases the recovery of Olsen P in all soils tested, except in those with very high Na content.

A recent investigation indicates that strong interactions between P_i _and humic materials is predicated on the presence of metal ions that act as cationic "anchors", allowing anionic humates and phosphates to associate [96]. Stability constants of humate-metal-P complexes tend to be high, with log K values in the range 4.87–5.92 (Zn- and Mg-anchor, respectively).

### Concluding remarks

Much has been learned about P mobility in calcareous media over the last five decades, but some gaps in understanding remain. Many of these occur at the molecular level of P interaction with subsurface species, including the detailed mechanism of P desorption under the influence of organic species. The role of humic materials in P mobilization is another area of research that has been given relatively little attention and is a potentially fruitful area of study. The use of humates as soil amendments presents an especially interesting case. The practice is gaining popularity – as borne out by the existence of more than 70 purveyors of these "nonconventional soil additives" in the U.S. alone [53] – but nothing is known about its environmental consequences.
